# Factors shaping the global political priority of addressing elder abuse: a qualitative policy analysis

**DOI:** 10.1016/S2666-7568(22)00143-X

**Published:** 2022-08

**Authors:** Christopher Mikton, Laura Campo-Tena, Yongjie Yon, Marie Beaulieu, Yusra Ribhi Shawar

**Affiliations:** a Demographic Change and Healthy Ageing, Department of Social Determinants of Health, Division of Healthier Populations, World Health Organization, Geneva, Switzerland; b Institute of Criminology, University of Cambridge, Cambridge, UK; c Implementation and System Transformation, Division of Country Health Policies and Systems, WHO Regional Office for Europe, Copenhagen, Denmark; d Université de Sherbrooke, Faculté des lettres et sciences humaines, École de travail social, Centre de recherche sur le vieillissement, CIUSSS Estrie-CHUS, QC, Canada; e Johns Hopkins University, Bloomberg School of Public Health, Paul H Nitze School of Advanced International Studies, Baltimore, MD, USA

## Abstract

**Background:**

Globally, 1 in 6 people aged 60 years and older experience elder abuse in the community annually, with potentially severe physical and mental health, financial, and social consequences. Yet, elder abuse remains a low global priority. We aimed to identify the factors accounting for the low global political priority of elder abuse.

**Methods:**

We systematically searched relevant peer-reviewed literature and organisational reports in multiple databases and interviewed 26 key informants in the field of elder abuse. We used policy frameworks developed by previous research into the determinants of the priority of global health issues, and a qualitative methodology to thematically analyse the literature and interviews through triangulation of the data.

**Findings:**

The main factors identified were related to the nature of the issue (the inherent complexity of elder abuse, pervasive ageism, insufficient awareness and doubts about prevalence estimates, and the intractability of the issue), the policy environment (the restricted ability in the field of elder abuse to capitalise on policy windows and processes), and the capabilities of the proponents of prevention of elder abuse (disagreements over the nature of the problem and solutions, challenges in individual and organisational leadership, and an absence of alliances with other issues).

**Interpretation:**

Around 25 years ago, elder abuse started to register on the global agenda. Since then, the global priority for prevention of elder abuse has barely increased. This study identifies several inter-related factors that account for the issue's low priority and opportunities for overcoming these challenges. Chief among these opportunities is the UN Decade of Healthy Ageing 2021–2030, a unique 10-year-long policy window to increase the political priority of the prevention of elder abuse.

**Funding:**

World Health Organization.

## Introduction

Globally, 1 in 6 people aged 60 years and older experience elder abuse in the community every year.^1^ Prevalence rates of elder abuse in long-term care facilities are higher still.^2^ The prevalence of elder abuse has increased during the COVID-19 pandemic.^3^

Elder abuse is defined by WHO as a single or repeated act or lack of appropriate action, occurring within any relationship where there is an expectation of trust, which causes harm or distress to an older person. Taking many forms, including physical abuse, psychological abuse, financial or material abuse, sexual abuse, and neglect,^4^ elder abuse is a public health, social, criminal justice, medical, and human rights issue that can have serious consequences, such as premature mortality, physical injuries, depression, cognitive decline, financial devastation, and placement in long-term care institutions.^5^, ^6^, ^7^ Data on global and national economic costs of elder abuse are scarce^8^ but, in the USA, elder financial abuse and fraud have been estimated to cost up to US$36·5 billion annually.^9^

Yet, despite its magnitude and severity, elder abuse remains a low global priority. Elder abuse receives little attention from international and national organisations and governments^10^, ^11^, ^12^, ^13^ and few resources.^11^, ^14^ For instance, in the USA, federal agencies' spending on elder abuse is equivalent to around 2% of their spending on violence against women.^15^ Global priority can be understood as the degree to which international and national political leaders actively give attention to an issue, and follow that attention with the provision of financial, technical, and human resources that are commensurate with the severity of the issue.^16^

Policy frameworks developed by previous research into the determinants of the priority of global health issues indicate that three key factors account for the global priority an issue receives:^16^, ^17^, ^18^, ^19^, ^20^ the nature of the issue; the policy environment; and the capabilities of proponents advocating for the issue. First, an issue is more likely to receive priority if it is straightforward; if the affected population is politically powerful, viewed sympathetically and not stigmatised, and is able to advocate for itself; and if the severity of the issue is clear, with tractable solutions available. Second, an issue is more likely to be prioritised if policy windows exist to advance the issue, including global goals, resolutions, and disasters (eg, the COVID-19 pandemic) highlighting the severity of the issue. Third, an issue is more likely to receive priority if proponents can agree on a common understanding of the problem and its solution (problem definition); frame the issue in a way that resonates with policy makers and donors (positioning); and if they can create cohesive and effective coordinating mechanisms, with effective leaders and champions to achieve collective goals (governance). Drawing on these frameworks, we aimed to identify the factors shaping the global political prioritisation of addressing elder abuse.

**Research in context**

**Evidence before this study**

We searched PubMed, MEDLINE, AgeLine, the International Bibliography of the Social Sciences, and Google Scholar between Oct 1 and Oct 15, 2020, without any language restrictions. Publications were included if they addressed elder abuse and determinants of global priority and were excluded if they did not address determinants of priority at the global level (eg, at national or subnational levels). The following terms and their cognates were used in various combinations in the searches: elder abuse (eg, elder mistreatment, elder neglect, older people, or violence), political priority (eg, priority, importance, visibility, agenda, or policy), and determinants (eg, factors and influences). These initial searches indicated that no previous study has investigated the factors accounting for the low global priority of elder abuse. The systematic search done for the study itself confirmed this finding. There was agreement in the literature that the issue of elder abuse globally was not receiving attention commensurate with the scale and severity of the problem. In a 2014 global survey to assess measures taken by countries to address interpersonal violence, elder abuse was consistently addressed the least often.

**Added value of this study**

To our knowledge, this study is the first of its kind to investigate the factors that account for the low global political priority of elder abuse. Drawing on social science literature and on a policy framework, we thematically analysed data collected from key informant interviews and from a systematic review of the literature using data triangulation. The study identifies factors connected to the inherent complexity of the issue, pervasive ageism, lack of awareness and doubts about prevalence estimates, the intractability of the issue, inability to capitalise on policy windows and processes, disagreements on the nature of the problem and its solutions, and weakness of governance structures.

**Implications of all the available evidence**

Many of the factors identified as accounting for the low global priority of elder abuse are amenable to change. The study discusses the main opportunities for effecting change and increasing the priority of elder abuse. Chief among these is the UN Decade of Healthy Ageing 2021–2030, which brings together governments, civil society, international agencies, professionals, academics, the media, and the private sector for 10 years of concerted action to improve the lives of older people, their families, and the communities in which they live, including by reducing elder abuse. The findings of this study will inform a strategy paper being developed for addressing elder abuse within the Decade of Healthy Ageing 2021–2030.

## Methods

### Study design

Our analysis was based on a systematic search for relevant peer-reviewed literature and organisational reports, of which 123 publications were included in a narrative synthesis, and on interviews with 26 key informants (table). Drawing on the key factors delineated in policy frameworks (nature of the issue, the policy environment, and the capabilities of proponents advocating for the issue) and using qualitative process tracing methodology,^21^ we thematically analysed^22^ the literature and the key informant interviews through triangulation of the data. To ensure complete reporting of our data collection and analysis, we followed the Consolidated Criteria for Reporting Qualitative Research.^23^ The policy frameworks were identified through a search for and an analysis of the literature on determinants of political priority of global health issues.^16^, ^17^, ^18^, ^19^, ^20^

**Table:** Description of informants in terms of type of organisation they work for, WHO region, and World Bank income level of country in which they reside

	**Informants (n=26)**
**Type of organisation**	
International governmental organisation	3
International non-governmental organisation	7
International non-governmental organisation and academia	9
Academia	5
Governmental organisation	2
**WHO region**	
African region	2
Region of the Americas	10
Eastern Mediterranean region	1
European region	8
South-East Asia region	1
Western Pacific region	4
**Country income level**	
High-income country	18
Upper-middle-income country	4
Lower-middle-income country	4
Low-income country	0

An application was submitted to WHO's Research Ethics Review Committee. The Committee exempted the application from review on the grounds that public officials were to be interviewed in their official capacity on issues that are in the public domain and that no information allowing them to be identified would be included in publications.

### Systematic review of the literature

We searched for relevant peer-reviewed and organisational reports, covering the period between Jan 1, 2000, and Jan 29, 2021, in the following databases: PubMed, MEDLINE, AgeLine, the International Bibliography of the Social Sciences, Global Health, ProQuest One Literature, JSTOR, WHO Global Health Library, Google Scholar, and several websites of organisations concerned with elder abuse (a full list is provided in the appendix pp 1–2). Searches took place from Nov 16, 2020, to Jan 29, 2021, and were mostly conducted in English, but also in Spanish and French in relevant databases. The full pre-planned search strategy is available in the appendix (pp 1–4).

Publications were included if they addressed elder abuse and at least one of the factors affecting the political priority of health issues (the full list is in the appendix pp 3–4). Publications were excluded if they only addressed the factors influencing political priority at a national or subnational level—rather than at a global level—or if they only focused on subtypes of elder abuse (eg, financial abuse) or on specific populations (eg, older people with dementia). The eligibility criteria were applied by two independent researchers (CM and LC-T). Disagreements were resolved through discussion.

The diversity of types of documents included in the review (eg, UN reports, policy documents, organisational reports, and scientific articles) precluded the assessment of their quality.

### Informant interviews

Using a purposive sampling strategy, we identified potential key informants in the field of elder abuse through the literature review, the research team's knowledge of key people in the field, and by asking informants themselves for suggestions of other informants. Potential informants were contacted using a standardised email and were assured that no personally identifying information would be published and that interview transcripts would remain confidential. Informants signed a consent form after reading an information sheet about the study and being given the opportunity to ask questions. We continued key informant interviews until we reached theoretical saturation, the point at which all major themes had been identified and additional interviews were unlikely to reveal new information.^24^

The interviews were done in English between Feb 22 and May 20, 2021, and lasted around 1 h. CM conducted seven interviews, LC-T conducted six interviews, YY conducted seven interviews, and MB conducted six interviews. Although each of the interviewers has expertise in the field of elder abuse or criminology, their focus varies (LC-T and MB are mainly research focused, while CM and YY also have a policy and advocacy focus) and each interviewer brought a different cultural and professional background to the interviews (CM and YY are research practitioners with WHO affiliation; MB and LC-T are academics with university affiliation). As much as possible, interviewers were matched with informants based on areas of expertise. For instance, interviewers with a research or policy background interviewed informants with similar backgrounds. Interviewers met regularly to discuss any problems that might have arisen and to ensure consistency in their approaches.

The semi-structured interview guide is available in the appendix (pp 5–9). The interviews were mostly conducted virtually via Microsoft Teams (Microsoft Corporation; Redmond, WA, USA) and Zoom (Zoom Video Communications; San Jose, CA, USA) with the exception of one written submission. With informants' permission, interviews were recorded and transcribed for analysis. Transcripts were not sent back to participants, but a draft of the manuscript was sent to all key informants to ensure that their confidentiality was not compromised.

### Data analysis

Drawing on the aforementioned policy frameworks and analytical approaches, we did a thematic analysis and narrative synthesis of the literature and the transcripts of the interviews. We started by coding deductively using a broad framework that included the nature of the issue, the policy environment, and proponent capabilities.^18^ The coding scheme was then iteratively and inductively developed to include themes that emerged through the triangulation of the data from the systematic review and key informant interviews. Relevant subcodes that were added included, for the nature of the issue, the inherent complexity of the issue, ageism, characteristics of the affected population, and prevalence and tractability of the issue; for the policy environment, policy windows and processes; and for proponent capabilities, subcodes for problem definition, its framing, organisational and individual leadership, and coalition building.

Four authors—CM, LC-T, MB, and YY—completed the coding, with the help of a coding sheet (appendix pp 10–11) and using Microsoft Word (Microsoft Corporation). Regular online meetings among coders and with YRS were held to ensure consistency of coding and to revise iteratively the coding sheet as subcodes emerged. Additionally, all coders checked each other's coding for consistency. The capital letter “I” followed by numbers presented in brackets—eg, (I1, I4, I28, etc)—indicate specific informants.

The focus of the analysis was on the political priority of elder abuse at the global level. We only focused on relevant documents, networks, actors, and debates occurring at a regional, national, or community level insofar as they affected global efforts to advocate for elder abuse.

### Role of the funding source

Two of the authors—CM and YY—work for WHO, which funded this study. The funder of the study had no role in study design, data collection, data analysis, data interpretation, or writing of the report.

## Results

### Systematic review findings

Of the 32 401 records identified, after removal of duplicates, 27 562 were screened by title and 26 529 (96%) were excluded (with 1033 remaining), a further 888 records were excluded based on abstracts (with 145 remaining), and a final 22 were excluded based on full-text articles (figure). Ultimately, 123 publications were included in the narrative synthesis. There was a high level of agreement between the reviewers (86·8%).

**Figure:**
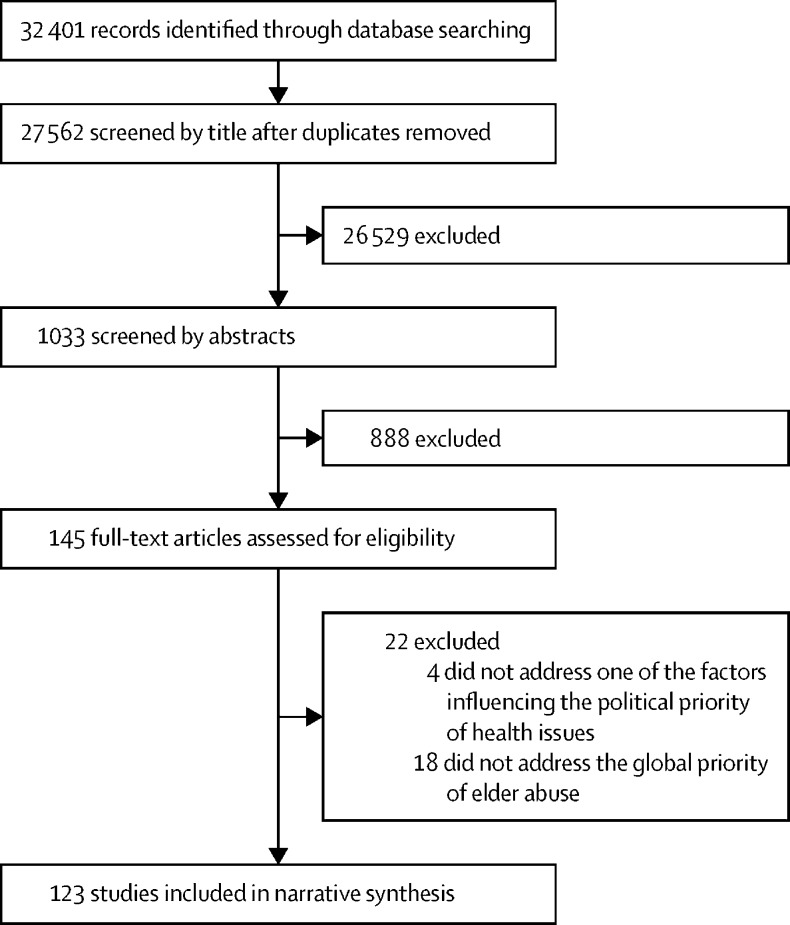
Flow diagram for systematic review

We contacted 29 potential informants and conducted 26 confidential interviews (90% response rate); 17 of the informants were female and nine were male, and 14 (54%) of the informants had more than two decades of experience working in the field of elder abuse. The types of organisations for which the informants work and the WHO regions and World Bank income level of the countries in which they are based are indicated in the table. Despite the efforts made to include informants from all regions and country income levels, most were from high-income countries. 21 (81%) informants were centrally involved in the field of elder abuse as experts, leaders, or advocates. The remaining five (19%) were considered informed onlookers, with extensive knowledge of the field by having spent their careers working in an adjacent field, for instance, as academics researching the prevention of interpersonal violence more broadly, in international organisations focusing on violence against women, or in civil society organisations addressing ageing.

### The nature of the issue

Most societies aspire, in theory at least, to treat older people with respect and sympathy and consider inflicting harm on them to be socially unacceptable. This should, according to the frameworks described in the Introduction, help to generate political priority for the issue.^18^ However, the analysis revealed that in practice things are less straightforward and four aspects of the nature of elder abuse complicate efforts to increase its priority.

#### Inherent complexity of the issue

Many informants reported that elder abuse is a particularly complex issue and more multifarious and complicated than many other issues (I1, I3, I6–8, I11–13, I15, I22, and I26), making it difficult for decision makers to grasp and act on. For instance, informants noted that it takes on markedly different forms, including physical, psychological, sexual, and financial abuse, as well as neglect and systemic or organisational abuse, and takes place both in the community and institutions. Inherent in elder abuse, they pointed out, is a tension between preserving the autonomy and self-determination of older adults and safeguarding those who are vulnerable and dependent (I3, I15–17, I19, and I23).^25^, ^26^, ^27^, ^28^ The different manifestations of elder abuse across cultures are also considered to pose a challenge to addressing it at a global level (I4, I16, and I23).^29^, ^30^, ^31^ Accusations of witchcraft or abandonment in hospitals or other institutions are, for instance, two distinct forms of elder abuse relevant to some contexts but not others (I2, I3, and I13–16).

#### Age, stigma, and shame

Ageism—stereotypes, prejudices, and discrimination based on age^32^—recurred as a theme in more than half of the interviews and in much of the literature.^26^, ^33^, ^34^ Many informants spoke with great passion about ageism being the principal cause of elder abuse and the *“major overall problem”*, accounting for the low priority of elder abuse (I3, I4, I6, I10, I11, I14, I16, I24, and I25). *“Older people are devalued and viewed as expendable”* (I23). In the literature, ageism was referred to as the key consideration to any policy targeting elder abuse.^35^ Some informants perceived elder abuse to be an extreme expression of ageism (I2, I3, I11, and I18).

The shame experienced by victims and their families, and the stigma associated with elder abuse in wider society might also impede elder abuse from receiving greater prioritisation (I3, I8, I13, I15, I16, and I25).^36^, ^37^, ^38^

*“Revealing this in the public space might not necessarily mobilize political attention but it might be perceived as shame and blame to certain members of the society.”* (I3)

#### Insufficient awareness of and doubts about the prevalence estimates

Global prevalence estimates of elder abuse might not be generating the expected attention for two reasons. First, awareness of these estimates is insufficient. Several informants, whose expertise was generally in policy rather than research, were unaware of these estimates (I3, I7, I11, and I15). Second, the credibility of these estimates has been questioned (I6 and I7). However, some informants believed that elder abuse remains under-reported (I8, I13, I15, and I26) and the literature suggests that, in both the community and institutional settings, older people experiencing abuse and their carers might not always understand what constitutes abuse, contributing to this under-reporting.^39^, ^40^ Another informant stated:

*“The only time [elder abuse] ever appears to be more than a low base-rate phenomenon is if people use wildly, inflated, and exaggerated indicators of psychological abuse.”* (I6)^27^

#### Intractability of elder abuse

The absence of effective interventions to address elder abuse acts as an impediment to the issue's advancement, as policy makers are more likely to prioritise issues that they believe they can act on. Reviews are almost unanimous in finding that, due to the generally low quality of studies, no clear conclusions about the effectiveness of interventions can be drawn and currently there are almost no interventions that have been proven to work in high-quality evaluations.^27^, ^41^, ^42^, ^43^, ^44^, ^45^ This conclusion was widely echoed among informants (I3, I4, I6–8, I10, I11, I13–15, I19, I21, I23, and I26), particularly researchers:

*“So, I think we need like the equivalent of…a Marshall Plan…the only thing that people ought to be funding or researching or doing as far as elder mistreatment now goes is intervention research.”* (I6)

However, informants who were not researchers often spoke as if they assumed that effective solutions existed, but without putting forward a set of agreed-upon interventions (I1, I5, I11, and I22).

However, a 2016 review identified several promising interventions. These included, for instance, caregiver interventions, which provide services to relieve the burden of caregiving (eg, housekeeping and meal preparation); telephone helplines, which allow older people to seek advice and assistance in case of abuse; and emergency shelters, which provide a safe place to escape from abuse and to make plans for guaranteeing safety in their homes.^27^

The doubts about prevalence estimates and the low quality of intervention studies reflect the underdevelopment, underfunding, and complexity of elder abuse research, emphasised both by informants and in the literature (I5–11, I13, I15, I16, I18–20, and I26):^8^, ^46^

*“[We] need mainstream public health researchers to become interested in [elder abuse] and use the full armamentarium of their methods.”* (I6)

### The policy environment

#### Elusive policy windows and processes

Proponents of the prevention of elder abuse have struggled to take full advantage of global policy windows and processes to raise the priority of elder abuse. Informants identified four important policy windows and processes which the field has, so far, failed to capitalise on sufficiently.

First, although the 2030 Sustainable Development Goals (SDGs) were identified as potentially useful (I2, I4, I7, I11, I15, I16, I22, and I26),^11^, ^30^ some informants pointed out that older people and elder abuse in particular are neglected within the SDGs, and elder abuse, unlike violence against women and children, has no SDG indicator of its own (I5 and I6).^47^

Second, many informants acknowledged that World Elder Abuse Awareness Day has helped raise awareness of the issue. However, several informants feared that the attention this day brings to elder abuse is fleeting with little impact, and that in recent years it has sometimes focused more on older people in general rather than on elder abuse specifically (I1, I14–20, I22, and I23).

*“I think these international days are good to raise awareness, but they only raise awareness and they don't have a major impact.”* (I16)

Third, although the UN Decade of Healthy Ageing 2021–2030 was viewed as presenting a major opportunity for raising the profile of the issue, informants reported that elder abuse had so far not figured prominently enough within it (I11, I13, I15, and I22).

Fourth, informants acknowledged that the COVID-19 pandemic has increased attention for elder abuse (I2, I3, I5, I7, I10, I16, I18, I19, and I21–26),^48^, ^49^, ^50^ particularly in long-term care facilities, but the attention remained insufficient and often did not translate into impactful polices (I1, I2, I12, I13, I19, and I24).

### Proponent capabilities

Four aspects of the capabilities of proponents against elder abuse also contribute to the low global priority of elder abuse.

#### Reaching a common understanding of elder abuse and the extent to which proven solutions exist

Although there is some convergence among proponents on a basic understanding of elder abuse (I1, I4–6, I10, I11, and I26), definitional wrangling continues, which might weaken the cohesiveness of the field and detract from advancing the issue. Disagreements centre around culturally specific forms of elder abuse (I1, I2, I8, I10, I13, I14, I16, and I23)—*“elder abuse is so different in different contexts”* (I3); how far the expectation of trust at the heart of the definition of elder abuse,^51^ should extend (eg, to strangers, financial institutions, and government; I7, I8, I11-13, I16, I18, I19, I21, I23, and I25); and the inclusion of self-neglect, financial fraud and scams, and systemic abuse within the definition (I10, I12–14, I16, I18–20, I22, and I23).^33^, ^51^ However, most importantly, for the development of effective solutions to be prioritised, the field has still to agree, as noted, on the extent to which proven solutions exist, if any.

*“We really haven't come very far in developing prevention and treatment options for [elder abuse].”* (I6)

#### Untapped synergies of a dual framing and the challenge of ageism

Potential synergies of the dual framing of elder abuse at the global level as both a human rights issue and a public health problem (I4, I5, I10, I13, I17, I19, I23, and I24)^11^, ^25^, ^52^, ^53^ have gone largely untapped.

Further strengthening the positioning of elder abuse as a human rights issue, in the hope that it will one day be included in a UN Convention on the Rights of Older Persons (I5, I11, I15, I16, and I26), was viewed, particularly by those working in international governmental and non-governmental organisations, as key to elder abuse receiving more attention (I1, I2, I10, I11, I13-16, I18, I19, I22, and I24).

Furthermore, informants thought that framing elder abuse as a wider violence prevention problem within a public health approach would also help garner more attention, as violence prevention, particularly the prevention of violence against women and children, is receiving more attention (I12, I18, I21, and I23).

The potential for these two framings to work synergistically to boost the issue of elder abuse has so far not been exploited. However, one informant noted:

*“I don't think they are mutually exclusive as a good public health framing includes human rights.”* (I4)^54^

#### Global networks and organisational and individual leadership

Global networks and organisational and individual leadership need strengthening. In the past 25 years, substantial progress has been made in establishing a global network to address elder abuse. Credit for this mainly belongs, the informants agreed, to the International Network for the Prevention of Elder Abuse (INPEA; I11, I12, I19, I20, and I22).

INPEA was the organisation most consistently identified as the main actor in this global network and on which informants focused most of their comments (I1, I3, I5, I6, I8, I10–12, I16, I18, I19, I21, I24, and I26).^30^, ^54^ Other organisations were also mentioned including—most often—WHO, HelpAge International (HAI), and the Office of the UN High Commissioner for Human Rights (OHCHR) and—less frequently—the UN Population Fund (UNFPA), UN Women, and the International Federation on Ageing. However, these other organisations were viewed as providing less consistent leadership and as addressing elder abuse more sporadically (I1–3, I5, I6, I11–13, I15, I17, and I26).

The informants praised INPEA's leadership and accomplishments in raising the priority of elder abuse globally by, for instance, becoming a non-governmental organisation with special consultative status at the UN (I10 and I12); bringing together diverse actors at global, regional, and national levels into an international network and pushing the issue of elder abuse in international fora (I8, I10, I11, I19, I20, and I26); and by increasing awareness of elder abuse, including by initiating the World Elder Abuse Awareness Day (I16, I18–20, I22, and I23).

Nonetheless, informants identified areas related to a lack of coordination and funding and to fragmentation, in which global networks need strengthening and which are impeding more effective collective action (I4, I9, I10, I12–15, I17, I19, I23, and I25). For instance, informants commented on the need for better mechanisms to coordinate activities between actors within the global network, such as WHO, HAI, OHCHR, UNFPA, and INPEA (I10 and I23).^14^, ^35^, ^55^ Insufficient funds, several informants pointed out, have hampered INPEA (I10, I11, I20 and I23). INPEA does not raise funds from governments to protect its independence, advocacy, and educational roles (I5).^56^ Several informants also referred to discord within INPEA (I2, I6, I9, I10, I17, I20, and I23). A point of contention was whether the network should take a broad approach in advocating for a UN Convention on the Rights of Older Persons, which would address elder abuse as one of many issues pertaining to older people, or whether it should focus more narrowly on elder abuse (I2 and I23).

Furthermore, no individual global leaders stand out clearly. Most informants identified many different individual leaders, but some identified none (I9, I21, I22, and I26). The absence of high-profile champions of elder abuse (I16, I17, and I25) was often attributed to ageism (I1, I3, I15, I19, I21, I23, and I25):

*“…everybody wants to stand up for women or stand up against sexual abuse…No one necessarily wants to be the poster boy or girl for elder abuse.”* (I21)

#### Need for more coalition building

A need for more coalition building was identified. Informants noted that significant alliances with external actors have not been forged to address elder abuse. Though there was agreement that such alliances should be a priority (I11, I19, I21, I22, and I24), there was less agreement on who among the potential allies should be prioritised. Competition for scarce resources (I1, I9, and I23), working in silos (I23), few elder abuse champions (I16, I17, and I25), and ageism (I1, I3, I19, I21, I23, and I25) were reasons given for the current lack of alliances.

The absence of a movement of older people against elder abuse or a movement of survivors of elder abuse was identified as a problem (I4, I11–16, I18, and I24–26).^57^

*“You don't have a strong older people's movement, [unlike with violence against women] where there are women's organisations and women's movements behind it [that are] relentless in pushing the issue.”* (I4)

Opinion was divided on forging alliances with the violence against women community (I1, I2, I6, I8, I11–13, I16, I19, I21, I23, and I26). Some informants thought that the communities were natural allies, given that most victims of elder abuse are women (I3, I13, I16, and I24). Other informants claimed that the violence against women community *“was the worst of the potential allies”* and *“only interested in younger women”* (I5 and I15); that it is too narrow an issue; and that strategically it would be better to build coalitions with issues higher up the global agenda and with more funding, such as ageism, disability, and dementia (I4, I13, and I17). The priority of ageism has recently received a boost by becoming one of the four action areas of the UN Decade of Healthy Ageing 2021–2030 and the focus of a Global Campaign to Combat Ageism.^58^

The human rights community was often viewed as one of the rare current or potential future allies (I5, I10, I11, I13-I16, I18, I19, I22, I23, and I26). Other potential allies mentioned were professional communities (eg, police force, human service professions, and physicians and, in particular, geriatricians; I3, I4, I9, I11, I12, and I20) and the broader violence prevention community (I17, I21, and I23).

## Discussion

This study identified key factors that shape the low global priority of elder abuse. The identified factors connected to the nature of the issue are the inherent complexity of the issue, pervasive ageism, insufficient awareness of and doubts about prevalence estimates, and the intractability of the issue. Factors related to the policy environment mainly concern proponents' restricted ability to capitalise on policy windows and processes. Factors relating to proponents' capabilities include disagreements on the nature of the problem and its solutions, challenges in individual and—especially—organisational leadership, and a dearth of alliances with other issues.

Over the past 25 years, progress has been made in putting elder abuse on the global agenda. For instance, in 1997, INPEA was founded. In 2002, the 2nd World Assembly on Ageing's Political Declaration and the Madrid International Plan of Action on Ageing included the elimination of all forms of neglect, abuse, and violence in relation to older people among its objectives. Also in 2002, WHO's landmark *World Report on Violence and Health*^25^ addressed elder abuse and the *Toronto Declaration on the Global Prevention of Elder Abuse*^59^ and WHO's study *Missing Voices: views of older persons on elder abuse*^28^ were issued. In 2010, World Elder Abuse Awareness Day became an officially recognised UN International Day. Yet, despite these advances, informants noted that in recent years progress seems to have stalled and elder abuse has struggled to achieve sufficient priority.

Currently, several opportunities exist for overcoming the challenges identified. First, the UN Decade for Healthy Ageing 2021–2030^58^ and the COVID-19 pandemic are two policy windows that the field can capitalise on. Findings from this study will inform a strategy paper for addressing elder abuse within the Decade of Health Ageing 2021–2030, to be published in 2022. Second, to strengthen coalition building, proponents could consider organising a series of coalition-building meetings under the auspices of the Decade for Healthy Ageing 2021–2030. Third, to strengthen coordination and leadership, the UN Inter-Agency Group on Ageing and the UN Decade of Healthy Ageing could consider, in collaboration with INPEA, creating a partnership of UN and other agencies to address elder abuse in a more concerted way and to appoint a lead agency within the UN system to coordinate efforts. Finally, the ongoing Global Campaign to Combat Ageism^60^ presents a major opportunity to address ageism, a key factor thwarting the prevention of elder abuse.

Making elder abuse more tractable might require the establishment of a global network to develop, evaluate, and scale up a package of effective and cost-effective interventions for elder abuse. The development of such packages for violence against women, called RESPECT,^61^ and for violence against children, called INSPIRE,^62^ appears to have reduced the sense of intractability of these other forms of violence and increased cohesion among the proponents of these packages.^17^ If it remains unclear which evidence-based solutions can be scaled up to reduce elder abuse, the field will continue to struggle to mobilise resources and the opportunity costs of addressing elder abuse might appear too high.

The ongoing disagreements about the definition of elder abuse point to the need for an updated and widely shared operational definition. Such a definition could also serve as a basis for selecting appropriate outcome measures in elder abuse intervention studies. Additionally, although only infrequently mentioned by informants (I5 and I21), making the economic case for the prevention of elder abuse could be an effective strategy for increasing its global priority.^18^

This study has some limitations. First, none of the informants were based in low-income countries, and fewer informants were based in middle-income countries than in high-income countries. Second, three of the WHO regions were markedly under-represented, namely the African region, the Eastern Mediterranean region, and the South-East Asian region. However, these two limitations can partly be explained by the fact that most global institutions for which many informants were working are based in high-income countries, particularly the region of the Americas or the European region. Third, only five (19%) of 26 informants worked for national or international governmental organisations. Thus, it is possible that some informants lacked first-hand experience of the competing interests and complexities involved in selecting priorities within such organisations.

In conclusion, around 25 years ago, elder abuse started to register on the global agenda. Since then, the prevention of elder abuse has struggled to increase in global priority. To increase the global priority of this issue, this study suggests that proponents of the prevention of elder abuse must overcome several inter-related challenges. Among the greatest of these challenges are reducing pervasive ageism, which contributes to the violence against and abuse of older people being taken less seriously than that experienced by other age groups; making the issue more tractable through the development of cost-effective solutions; and developing more cohesive and effective structures of governance. The UN Decade of Healthy Ageing 2021–2030 presents a unique 10-year policy window to take on—and overcome—the challenges facing the field.

## Data sharing

Transcripts of the interviews cannot be made available as informants were guaranteed confidentiality and anonymity. The content of the transcripts would, in several cases, almost certainly allow the identity of the informant to be ascertained. However, further details about the searches for the systematic review will be made available on the platform of the UN Decade of Healthy Ageing.

## Declaration of interests

We declare no competing interests.
