# Supplementary material for: Factors shaping the global political priority of addressing elder abuse: a qualitative policy analysis
Source: Lancet Healthy Longev. 2022 Aug;3(8):e531–9. doi: 10.1016/S2666-7568(22)00143-X (PMC9360496; doi:10.1016/S2666-7568(22)00143-X)
Supplement: Supplementary appendix [file mmc1.pdf]

THE LANCET
Healthy Longevity

Supplementary appendix

This appendix formed part of the original submission and has been peer reviewed. We post it as supplied by the authors.

Supplement to: Mikton C, Campo-Tena L, Yon Y, Beaulieu M, Shawar YR. Factors shaping the global political priority of addressing elder abuse: a qualitative policy analysis. *Lancet Healthy Longev* 2022; published online July 8. https://doi.org/10.1016/S2666-7568(22)00143-X.

Supplementary Material

1. Full search strategy for literature review for "Understanding the factors shaping the global political priority of addressing elder abuse"

Databases (mainly peer-reviewed literature):

- PubMed/Medline
- AgeLine
- International Bibliography of the Social Sciences
- Global Health (https://www.ebsco.com/products/research-databases/global-health)
- ProQuest One Literature
- JSTOR (CRM has access to JSTOR multidisciplinary Arts and Sciences Collections I,II,III,V,VII, VIII and XIV. This incorporates access to a scholarly archive of over 1,000 full-text electronic journals in the following fields: history, economics, archaeology, classics, sociology, Asian, African, Latin American, Middle Eastern and Slavic studies, political science, music, film studies, performing arts, mathematics, ecology, psychology, business, art, architecture, language, literature, law, religion, and public policy.)
- WHO Global Health Library (LILACS, IBCS, BDENF, African Index Medicus, Index Medicus for Eastern Mediterranean Region (IMEMR), Latin American and Caribbean Center on Health Sciences Information (PAHO) Library, Western Pacific (WPRO)

Websites

- Google
- Google Scholar
- Websites of key organizations
  - World Health Organization: https://www.who.int/
  - UNDESA: https://www.un.org/en/desa or https://www.un.org/development/desa/ageing/
  - OHCRH: https://www.ohchr.org/EN/pages/home.aspx
  - UNFPA: https://www.unfpa.org/
  - UN women: https://www.unwomen.org/en
  - UN-ECE: https://www.unece.org/info/ece-homepage.html
  - World Bank: https://www.worldbank.org/
  - Inter-American Development Bank: https://www.iadb.org/en/about-us/overview
  - Global Alliance of National Human Rights Institutions (GANHRI): https://ganhri.org/
  - Regional NHRI networks and the cross regional fora African Union: https://ijrcenter.org/national-human-rights-institutions/regional-nhri-networks-and-forums/
  - EU: https://europa.eu/
  - Age Platform Europe: https://www.age-platform.eu/
  - INPEA: http://www.inpea.net/
  - ASEAN: https://asean.org/
  - African Union: https://au.int/
  - HelpAge International: https://www.helpage.org/
  - Global Alliance for the Rights of Oder People: https://www.eldis.org/organisation/A66465
  - International Federation on Ageing: https://ifa.ngo/
  - International Longevity Alliance: http://longevityalliance.org/
  - NGO Committee on Ageing: http://www.ngocoa-ny.org/
  - International Association of Gerontology and Geriatrics: https://www.iagg.info/
  - AARP International: https://www.aarpinternational.org/
  - US Centers for Disease Control and Prevention: https://www.cdc.gov/
  - US National Centre for Elder Abuse: https://ncea.acl.gov/

+ Suggestions experts volunteer during interviews

Search terms (also translated in French and Spanish):

A. Older adults: older adults, older people, frail elderly, aged, elderly, seniors
B. Elder abuse: elder abuse, elder neglect, elder mistreatment, elder maltreatment
C. Violence & abuse (A & C): abuse, domestic violence, intimate partner violence, abuse, violence, aggression, crimes, harmful behaviour, anger, rape, hostility, conflict, verbal abuse, physical abuse, sexual abuse, emotional abuse, psychological abuse, financial abuse, exploitation, fraud, assault
D. Institutions (C & D): Nursing homes, assisted living, residential care institutions, residential facilities, health facilities, skilled nursing facilities.
E. Determinants of political priority: agenda, assessment, evaluation, strategy, plan, proposal, governance, network, policy, priority, importance, institution, organization, workforce, burden, capacity, perception, cost, cost-effectiveness, leadership, frame/framing, institutions, community, mobilization, metrics, measurement, data, indicators, evidence, intervention, network, champions, mandate, definitions, resources, advocacy, support, campaign, global, international, developing countries, low-income countries, middle-income countries, implementation, action plan.

Inclusion criteria

Dates

- from 2000 till January 2021;
  - We were interested in the current and recent determinants of the global political priority of elder abuse, so going back before 2000 would be unnecessary.

.

Languages

- No language restrictions

Subject areas

- Addresses elder abuse and one of the 4 categories or 11 factors influencing the political priority of public health issues at global or larger regional level (e.g., a WHO region or in some larger regional entity such as the EU, ASEAN, etc.) below [1]:

Table 1. *The four categories for the framework on determinants of political priority for global initiatives.*

	Description	Factors shaping political priority
Actor power	The strength of the individuals and organisations concerned with the issue	1 Policy community cohesion: the degree of coalescence among the network of individuals and organisations that are centrally involved with the issue at the global level<br>2 Leadership: the presence of individuals capable of uniting the policy community and acknowledged as particularly strong champions for the cause<br>3 Guiding institutions: the effectiveness of organisations or coordinating mechanisms with a mandate to lead the initiative<br>4 Civil society mobilisation: the extent to which grassroots organisations have mobilised to press international and national political authorities to address the issue at the global level
Ideas	The ways in which those involved with the issue understand and portray it	5 Internal frame: the degree to which the policy community agrees on the definition of, causes of, and solutions to the problem<br>6 External frame: public portrayals of the issue in ways that resonate with external audiences, especially the political leaders who control resources
Political contexts	The environments in which actors operate	7 Policy windows: political moments when global conditions align favourably for an issue, presenting opportunities for advocates to influence decisionmakers<br>8 Global governance structure: the degree to which norms and institutions operating in a sector provide a platform for effective collective action
Issue characteristics	Features of the problem	9 Credible indicators: clear measures that show the severity of the problem and that can be used to monitor progress<br>10 Severity: the size of the burden relative to other problems, as indicated by objective measures such as mortality levels<br>11 Effective interventions: the extent to which proposed means of addressing the problem are clearly explained, cost effective, backed by scientific evidence, simple to implement, and inexpensive

And/or:

Addresses on of the three categories or 10 factors which influence network emergence and effectiveness at global level in table below. But focus primarily on the three categories.

These are:
(1) features of the networks and actors that comprise them, including leadership, governance arrangements, network composition and framing strategies;
(2) conditions in the global policy environment, including potential allies and opponents,
funding availability and global expectations concerning which issues should be prioritized; and
(3) characteristics of the issue, including severity, tractability and affected groups.

But see table below for more detail[2] .

Table 2. *Factors that may influence network emergence and effectiveness and presumed direction of causality.*

Category	Factor	Presumed direction of causality	Explanation	Example
Network and actor features	1. Leadership	+	A network is more likely to emerge and be effective if capable, well-connected and widely respected champions are available to lead the cause.	Strong leadership for neglected tropical diseases facilitates network emergence, attention and funding in past decade.
	2. Governance	+	Networks are more likely to be effective if they have appropriate governing structures capable of facilitating collective action and resolving disputes.	Absence of guiding institutions on pneumonia contributes to neglect of disease until mid-2000s.
	3. Composition	+/−	Networks that link diverse actors are more likely to generate creative solutions to problems but also to be hampered by disagreements.	Alcohol network dominance by scientists limits advocacy efficacy; diversity in TB network leads to disagreements over goals.
	4. Framing strategies	+	Networks are more likely to be effective when their members have discovered ways of positioning the issue that resonate with external actors, especially political elites.	Positioning of TB as an emergent threat to citizens of industrialized societies sparks global action in late 1980s.
Policy environment	5. Allies and opponents	+/−	Groups with aligned interests will facilitate network expansion and power. Opponents will challenge network legitimacy and issue promotion, but their existence may inspire mobilization.	Tobacco industry lobbying may both limit network effectiveness and also inspire more mobilization.
	6. Funding	+/−	Donor funding may facilitate network emergence and effectiveness and a dearth may hinder prospects for sustainability, but over-reliance on these resources may hamper network legitimacy.	Emergence, decline and resurgence of global network committed to provision of family planning services.
	7. Norms	+/−	Widely held expectations that global actors address a particular condition facilitate network emergence. Networks that advocate for policies that violate strong social values face obstacles.	Maternal survival network strengthens following inclusion of issue in MDGs, but remains reluctant to take on safe abortion.
Issue characteristics	8. Severity	+	Network emergence and effectiveness are more likely surrounding problems that are perceived to have high mortality, morbidity or socioeconomic costs.	Data on malaria's disease burden and disruptive economic effects facilitate network emergence in 1950s and again in 1990s.
	9. Tractability	+	Networks are more likely to form and be effective surrounding problems for which solutions exist or are perceived to exist, especially if proposed solutions are politically uncontroversial.	Evidence on efficacy of community-level interventions facilitates expansion of newborn survival network.
	10. Affected groups	+	Network emergence and effectiveness are more likely on issues that affect groups that are readily identifiable, that societies view sympathetically, and that are able to advocate for themselves.	Networks stronger to address HIV/AIDS from MTCT than from intravenous drug use; PLWHA a backbone for AIDS advocacy.

Exclusion criteria

- Addresses one of these categories or factors, but at national level or smaller sub-regional level (e.g., small number of countries);
- Focuses on sub-types of elder abuse (e.g., violence against older women, sexual violence, financial abuse) or on specific populations (e.g., older people with cognitive impairment).

References

1 Shiffman, J. & Smith, S. Generation of political priority for global health initiatives: a framework and case study of maternal mortality. *Lancet* **370**, 1370-1379, doi:10.1016/S0140-6736(07)61579-7 (2007).

2 Shiffman, J. *et al.* A framework on the emergence and effectiveness of global health networks. *Health Policy Plan* **31 Suppl 1**, i3-16, doi:10.1093/heapol/czu046 (2016).

2. Key informant interview guide:

Understanding the factors shaping the global political priority of addressing elder abuse

Text in italics NOT to be spoken

Non-italic text in bold – core questions asked of all

Thank you again for participating in this discussion to help us understand the priority of elder abuse in the global agenda– I very much appreciate the time you are taking to speak with me.

Before we start, we thought it would be helpful to provide a brief background on our study.

The purpose of this study is to gain a better understanding and explore more systematically the factors behind the inadequate global political priority of elder abuse and the challenges that need to be addressed in order to increase attention to this area. This interview will help us do this.

The analysis aims to explore 4 general areas:

1) The composition of the networks and relationships among the actors involved addressing elder abuse;

2) Their understanding of the nature of the problem and solutions;

3) The efficacy of the public framings they have used to attract political support; and

4) Factors connected to the policy environment and the nature of the issue itself that shape priority.

We hope to get your insights into each of these 4 areas.

Do you have any questions for me before we begin?

Great, and before we begin, one important note I'd like to emphasize, this interview is completely confidential, meaning your name will not be associated with anything you share with me today.

And so with that, I am wondering if I may have your permission to record our conversation to supplement my note-taking?

Thank you. Would you please confirm that you consent to participate in this interview, which will remain completely confidential?

Questions

Checklist:

1. Definition of problem and solutions
2. Composition proponent network/community
3. Cohesion of network/community
4. Leadership
5. Coalition – allies & opponents
6. Framing/positioning
7. Issue characteristics
8. Policy-political environment
9. Funding

<u>*Broad Introductory Questions – Level and Evolution of Priority*</u>

- **I use the term "elder abuse" – which includes neglect – during this interview, although I recognize that others use different words to describe this problem/issue. What terminology do you use and why?**
  - What do you understand it to encompass?
  - Do you think current definitions of elder abuse adequately cover the phenomenon of elder abuse as it manifests itself across different settings (such as the community and institutions) and cultures?
  - In your opinion, who has been involved in defining the issue?
  - Do you think it makes up a distinctive field? And, if so, when did it emerge as a field?

- *Level of prioritization*: **In your opinion, to what extent is elder abuse a global priority[1]?**
  - On a scale of 1-10, to what extent do you think it is prioritized at the global level?

- **What evidence would you cite to back your perspective?**
  - *History*: Has the global priority of elder abuse changed over time, especially over the last 20-30 years?
  - If so, how would you say it has changed?
  - Are there particular developments that mark shifts in global prioritization of the issue?

<u>**Actors/Networks Concerned about elder abuse**</u>

I. Problem Definition

- **To what extent are proponents of elder abuse speaking with one voice?**
  - **To what extent is there consensus on or major disagreements about what <u>the problem is and how it should be understood?</u>**
  - How well are proponents able to manage and transcend differences?
  - To what extent do you think problems defining elder abuse have hampered advocacy efforts?
- **To what extent is there consensus on the solutions to the problem of elder abuse?**
  - In your opinion, what is the best investment – in terms of financial and human resources – to prevent/address elder abuse?

II. Governance and coalition-building

[1]Degree to which global organizational and national govt leaders actively pay attention to an issue and provide resources commensurate with the problem's severity.

Checklist:

10. Definition of problem and solutions
11. Composition proponent network/community
12. Cohesion of network/community
13. Leadership
14. Coalition – allies & opponents
15. Framing/positioning
16. Issue characteristics
17. Policy-political environment
18. Funding

- *Proponent Community/Network:* **Which individuals and organizations make up the network of actors and policy community concerned with elder abuse? Please describe the key actors that make up this issue area.**
  - What are their professional identities? What are the major sectors involved (i.e., geriatrics, social work, psychology, law enforcements, etc.)?
  - To what extent are they from high vs. low- and middle-income countries?
    - To what extent are they integrated in or part of higher-levels of international organizations and the UN system?
    - *Grass-roots involvement*: To what extent have policy community members incorporated and created opportunities for grassroots actors to lead and have their voices heard?
    - *Opposition:* Which actors, if any, present the greatest opposition to advancing the elder abuse agenda?
      - o What form does their opposition take and what justifications to they put forward for it?
      - o And how well have proponents addressed this opposition?

- *Cohesion of community*: **To what extent do those concerned with elder abuse constitute a cohesive policy community?**
  - What are the major points of agreement/disagreement?
  - What impact has their cohesion/lack of cohesion had on the field?

- **Leadership**
  - o *Individuals*: **Are there individual champions/leaders who have been able to unify advocates, leading the charge for preventing/addressing elder abuse?**
    - What has made them an effective leader(s)? OR why do you think there has been a lack of leadership for this issue?
  - o *Orgs/Entities:* **In your opinion, are there organizations that are considered leaders in championing this issue at the global level?**
    - What has made them an effective leader(s)? OR why do you think there has been a lack of leadership for this issue?
    - *Governance/Coordination:* What global guiding institutions exist for coordinating collective action to advance the elder abuse prevention agenda? How effective have these institutions been?
- *Specific Organizations/entities who are leading the field* **(***identified in Question 5 above)*
  - How did X come about? To what extent has X prioritized addressing elder abuse? (i.e., X= WHO, etc.).
  - How effective has X been in bringing attention and resources to the issue? (i.e., X=WHO, etc.).
  - *The organizations/affiliated with respondent:* To what extent has your organization prioritized older abuse?
  - *National-level: Coordination across sectors:*
    - o Within the government in the country you know best, what ministry is responsible for programming and policies for addressing elder abuse?
    - o What are the national goals related to elder abuse and who sets them?

- How are policies and programs coordinated across ministries/sectors for elder abuse?
- As far as you known, is this consistent across countries?

Checklist:

1. Definition of problem and solutions
2. Composition proponent network/community
3. Cohesion of network/community
4. Leadership
5. Coalition – allies & opponents
6. Framing/positioning
7. Issue characteristics
8. Policy-political environment

- *Coalition-building*: **To what extent have elder abuse prevention proponents forged alliances with actors concerned with other issue areas?**
  - (For example, healthy ageing, ageism, human rights, and violence against women. To what extent have proponents engaged with or built formal or informal relationships with proponents in these areas? To what extent is elder abuse separate from these other agendas?)
  - *Violence against women* – have there been a strong alliance between VAW and elder abuse, two fields with many potential overlaps between the fields? If not, why have these alliances not arisen?
  - *Opposition:* To what extent has there been push-back to incorporating elder abuse prevention by various constituencies (for example, social services, the medical community)? Why do you think they are reluctant to take on elder abuse issues?

III. Framing/Positioning

- **How have proponents made the case for addressing elder abuse to leaders of international institutions and key stakeholders?**
  - How effective have they been in shifting priorities within these institutions and key national governments?
  - How well have proponents conveyed the severity of the problem?
  - How convincing are the solutions that proponents have proposed, and how effectively have they communicated solutions as a means of attracting political support?

Issue characteristics

- What characteristics of the problem make it more difficult to generate action?
  - Has the fact that elder abuse occurs both in the community and in institutions (such as care homes) had a bearing on generating action?
- What characteristics of the problem facilitate attention to the issue?
  - To what extent are there data/research gaps or measurement difficulties in this area?
  - And are there problems of access to the data at this time? Which important actors would you say have trouble accessing these data?

Policy/Political environment

- How have global declarations and goals shaped attention to the issue, including World Health Assembly resolutions, the SDGs, and World Elder Abuse Awareness Day?
- Has the COVID-19 pandemic shaped attention to elder abuse and, if so, how?
- What other developments have shaped attention to elder abuse—for instance, seminal studies, intervention alternatives, and social movements?
- To what extent are there global accountability mechanisms for ensuring elder abuse is addressed? Who is responsible for compliance?

Funding

- Do you think the funding for addressing elder abuse globally is adequate?
- Which areas in particular require more funds? (e.g., research, programme scale up, advocacy, network building).

Checklist:

1. Definition of problem and solutions
2. Composition proponent network/community
3. Cohesion of network/community
4. Leadership
5. Coalition – allies & opponents
6. Framing/positioning
7. Issue characteristics
8. Policy-political environment

Last Questions

- **In your opinion, what has been the biggest challenge in raising elder abuse prevention as a priority at the global and national levels?**
- **In your opinion, what needs to be done to raise this issue as a global priority?**

Opportunities for participants to ask questions of their own

- I have now come to the end of our questions, is there anything you would like to ask me about this study?

3. Coding sheet used for analyses

Domain	Theme	Codes	Dimensions along which theme can be evaluated	Definition/comments
Nature of the issue	Inherent complexity	IC	High-low complexity	Extent to which nature of elder abuse itself is complex (i.e. multi-dimensional).
	Characteristics of the affected population	AP	Easily identifiable – not identifiable<br>Viewed sympathetically – not viewed sympathetically<br>Able – unable to advocate for itself	Extent to which affected group is a readily identifiable group, which societies views sympathetically, and which is able to advocate for itself.
	Prevalence	P	Availability of data: high – low<br>Quality of data: high-low	Extent to which data are available – and quality of data - to show the prevalence and severity of the problem that can be used to monitor progress.
	Tractablity	T	High – low	Extent to which solutions exist or are perceived to exist, including extent to which proposed solutions are politically uncontroversial.
	Ageism	A	Prominence of ageism as issue: high -low	Ageism as an explanation for the low priority of elder abuse and links and overlaps between ageism and elder abuse.
Policy environment	Policy windows and processes	PWP	Many important windows and process – few windows and process<br>High – low capacity to capitalize on policy windows	..
Proponents capabilities	Problem definition	PD	Cohesive (i.e. high degree of agreement) – contested	Extent of consensus on both the concept (words used), problem and its solutions)
	Problem framing	PF	Cohesive (i.e. high degree of agreement) – contested	Portrayal that aims to resonate with and inspire external audiences and political leaders to act.
	Organizational leadership	OL	Strong – weak;<br>Unified – fragmented;	The effectiveness of organizations or coordinating mechanisms with a mandate to lead the initiative; Establishing institutions, particularly global institutions, to facilitate collective action. Think of most the important/most recent resolution, strategy, action plan, etc. and most important organizations, partnerships, alliances, networks, forums, commissions, etc.
	Individual leadership	IL	Strong – weak;<br>Unified – fragmented;	The presence of individuals capable of uniting the policy community and acknowledged as particularly strong champions for the cause.<br>List leaders mentioned in interview.
	Coalition-building	DB	Frequent – rare<br>Coalitions: narrow – broad	Forging alliances with external actors, particularly ones outside the health sector. Opponents will challenge network legitimacy and issue promotion, but their existence may inspire mobilization.<br>List key actors mentioned in interview.
Other themes	Challenges	C	N/A	Challenges identified by interviewee to increasing the priority of elder abuse.
	COVID-19	CV	N/A	Impact of COVID-19 pandemic on elder abuse.
Community vs. Institution	Community vs institutions	COI	N/A	EA in the community or in institutions.
Gender lens	Gender lens	GL	N/A	Gender as issue in relation to EA.
HIC vs LMIC	High vs LMICs	HLMIC	N/A	EA in high vs low- and middle-income countries.
History of priority	History of priority of elder abuse	HP	N/A	This concerns the history of the priority of EA and NOT the history of EA.

Explicit reasons for lack of priority	Explicit reasons given for lack of priority	ER	N/A	These are reasons explicitly given by interviewee explaining why EA has insufficient global priority. Not possible factors for its inadequate priority inferred by us.
Media portrayal	Media portrayal of EA	MP	N/A	Mention of portrayals of EA in media.

Abbreviations. EA: Elder abuse; HIC: High-income country; LMIC: Low- and middle-income country.
